# 

**Published:** 1883-07

**Authors:** 


					﻿JULY, 1883.
^X^HIRTIETH year.
HALL’S
JOURNAL
OF
HEALTH
; Guaranteed Circulation 200,000 Copies in 12* Months.
E. H. GIBBS, A.M., M.D., Editor,
No. 21 CLINTON PLACE, EIGHTH STREET, NEW YORK.
P TERMS : $1 a Tear.
Single numfier, 10 ctsX
				

